# Crystal structure of the thermochromic bis­(di­ethyl­ammonium) tetra­chlorido­cuprate(II) complex

**DOI:** 10.1107/S2056989015023348

**Published:** 2016-01-01

**Authors:** Emily P. Aldrich, Katherine A. Bussey, Jennifer R. Connell, Erin F. Reinhart, Kayode D. Oshin, Brandon Q. Mercado, Allen G. Oliver

**Affiliations:** a Department of Chemistry & Physics, Saint Mary’s College, Notre Dame, IN 46556, USA; b Department of Chemistry, Yale University, New Haven, CT, 06520, USA; c Department of Chemistry & Biochemistry, University of Notre Dame, Notre Dame, IN 46556, USA

**Keywords:** crystal structure, four-coordinate copper(II) complex, thermochromism

## Abstract

In the structure of the title complex salt, (Et_2_NH_2_)_2_[CuCl_4_], the asymmetric unit consists of four unique di­ethyl­ammonium cations and three unique tetra­chlorido­cuprate anions. Two of the three anions are located with their copper atoms on independent crystallographic twofold axes, while the remaining tetra­chlorido­cuprate is located at a general position of the ortho­rhom­bic space group *P*2_1_2_1_2. Two of the three Cu atoms adopt a distorted square-planar/disphenoidal geometry and the third Cu atom has a regular square-planar coordination environment. The di­ethyl­ammonium cations form an extensive hydrogen-bonded network through N—H⋯Cl inter­actions with the tetra­chlorido­cuprate anions, resulting in a two-dimensional sheet-like hydrogen-bonded network parallel to the *ab* direction. The complex was observed to undergo a color shift from deep green at room temperature to pale yellow at temperatures above 328 K.

## Abstract 2

The low-temperature structure of bis­(di­ethyl­ammonium) tetra­chlorido­cuprate is reported. The complex exhibits thermochromism and has a two-dimensional hydrogen-bonded network through N—H⋯Cl hydrogen bonds.

## Chemical context   

Thermochromic compounds exhibit a reversible change in color corresponding to a change in temperature. This change can occur in the solid state or in solution and is typically due to geometry rearrangement at the mol­ecular level. Several mechanisms have been proposed for this rearrangement, including phase transitions, changes in solvation, changes in ligand geometry, coordination number, and finally crystal packing (White & LeBlanc, 1999 ▸). There are two generally accepted classes of thermochromism: (i) continuous; used to describe a gradual change in color, most likely due to breaking or rearrangement of the crystal structure (Roberts *et al.*, 1981 ▸), and (ii) discontinuous; used to describe a dramatic change in color over a specific or small temperature range (Van Oort, 1988 ▸). Two classes of thermochromic compounds that have practical applications today include liquid crystals and leuco dyes. Liquid crystals exist on the boundary between the liquid and solid states. They are classified as discontinuous due to the chemistry of their transitions (Amberger & Savji, 2008 ▸). As a result, thermochromic liquid crystals have been used to make ‘mood rings’, thermometers, and game pieces (Chandler, 2012 ▸). Although color changes in liquid crystals are more sensitive to external stimuli such as temperature changes, they have a highly specialized manufacturing process and are difficult to make. For this reason, new thermochromic compounds such as leuco dyes are highly sought after. Leuco dyes are easier to work with and less sensitive to temperature changes. They have been used in advertising labels, textiles, and packaging for microwaveable syrup bottles and beverage cans that indicate content temperature changes (Muthyala, 1997 ▸). Given the intriguing applications of thermochromic compounds, we report the synthesis and structural characterization of a bis­(di­ethyl­ammonium) tetra­chlorido­cuprate complex (I) that displays thermochromic properties.
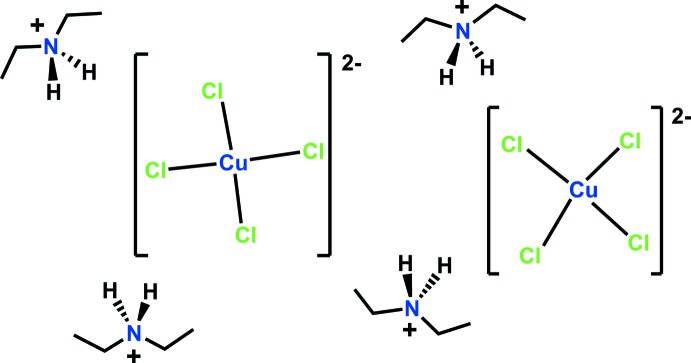


## Structural commentary   

**Figure 1:**
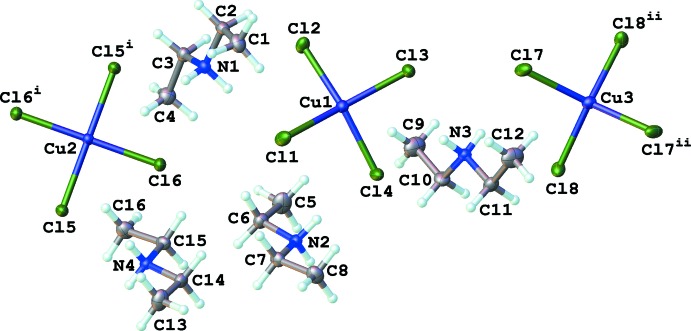
Atom labelling scheme for bis­(di­ethyl­ammonium) tetra­chlorido­cuprate. Atomic displacement ellipsoids are depicted at the 50% probability level and H atoms as spheres of an arbitrary radius. [Symmetry codes: (i) −*x* + 1, −*y* + 1, *z*; (ii) −*x* + 1, −*y*, *z*.]

The asymmetric unit of the thermochromic complex (Et_2_NH_2_)_2_[CuCl_4_] consists of four unique di­ethyl­ammonium cations and one full and two half tetra­chlorido­cuprate anions (Fig. 1 ▸). The di­ethyl­ammonium cations and the complete anion (Cu1) occupy general positions within the unit cell. The two half-tetra­chlorido­cuprate anions are located on crystallographic twofold axes at [½, ½, *z*] and [½, 0, *z*]. Each copper cation exhibits different coordination geometries. Cu2, located on a twofold rotation axis, has close to ideal square-planar geometry, with *trans* Cl—Cu—Cl angles close to 180° (Table 1 ▸). Analysis of these angles through the τ_4_ metric developed by Yang *et al.* (2007 ▸) yields a τ_4_ value of 0.02 for Cu2. A value of zero (0) is indicative of an ideal square-planar geometry while a value of one (1) indicates an ideal tetra­hedral geometry. In contrast, Cu1 and Cu3 adopt distorted square-planar geometries, tending to a disphenoidal (or ‘see-saw’) type geometry with τ_4_ = 0.27 and 0.48, respectively. The τ_4_ value is calculated from: [360 − (α + β)]/141; where α and β are the two largest angles about the four-coordinate copper(II) atom in question. However, these distortions are solely in the bond angles about the copper(II) atoms: all of the Cu—Cl bond lengths are similar (Table 1 ▸). A mean-plane analysis of each copper(II) atom shows the gradual change from the atoms being nearly co-planar (Cu2), through an inter­mediate distortion (Cu1) to a more pronounced out-of-plane arrangement of chlorine atoms around Cu3, in which the chlorine atoms are located 0.68 Å from the mean plane (Table 2 ▸). These distortions, along with the hydrogen-bonded network described below, are likely the cause for the thermochromism observed within the sample.

## Supra­molecular features   

**Figure 2:**
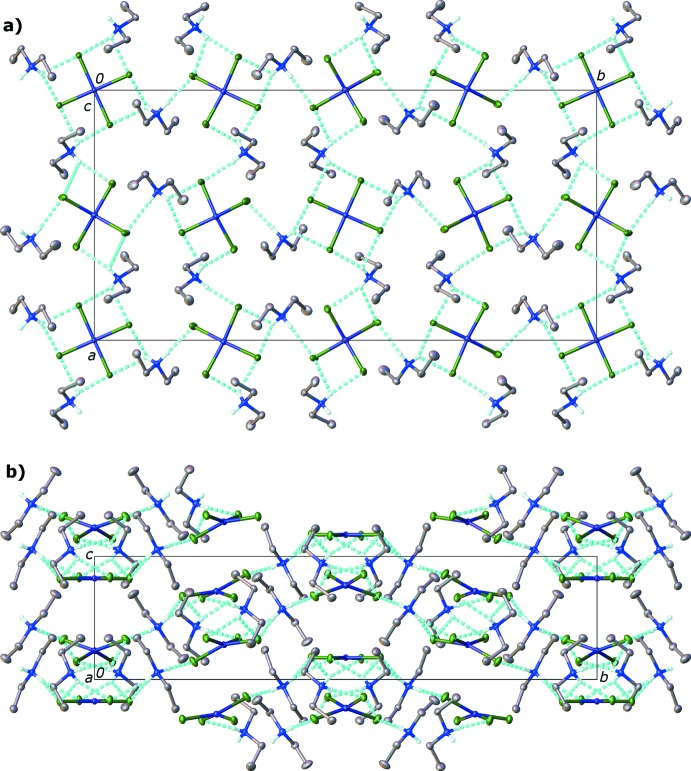
Hydrogen-bonding scheme for bis­(di­ethyl­ammonium) tetra­chlorido­cuprate viewed (a) along the *c* axis and (b) along the *a* axis. Atomic displacement ellipsoids are depicted at the 50% probability level and H atoms as spheres of an arbitrary radius. Ethyl H atoms have been omitted for clarity. Hydrogen bonds are shown as blue dashed lines.

The extended structure consists of the di­ethyl­ammonium cations forming an extended hydrogen-bonded network with the chlorine atoms of the tetra­chlorido­cuprate anions. All of the ammonium cations serve as hydrogen-bond donors; the ammonium cation hydrogen atoms were located in difference Fourier maps and refined freely. Ammonium cations involving N1, N2 and N3 all serve as donors of a single hydrogen-bond to one chlorine and as a donor of a bifurcated hydrogen bond to a pair of chlorine atoms on one copper(II) atom. The hydrogen atoms on N4 both form bifurcated inter­actions, albeit weakly (Table 3 ▸). All of the chlorine atoms serve as hydrogen-bond acceptors (Table 3 ▸, Fig. 2 ▸). While some of the reported inter­actions are quite long (N⋯Cl > 3.2 Å), and could be classified as weak inter­actions (Jeffrey, 1997 ▸), they are observed where the hydrogen atom is inter­acting with two chlorine atoms that are adjacent to each other/bonded to the same copper (II) atom and are considered by us to be bifurcated hydrogen bonds.

The Cu2 anion is notable because all four chlorine atoms are acceptors of bifurcated hydrogen bonds from N1 and N4; Cu2 is located on a twofold rotation axis. N1 also donates a single hydrogen bond to Cl1. N2 has a bifurcated hydrogen bond to chlorine atoms Cl2 and Cl3 on Cu1 and also forms a single donor hydrogen bond to Cl4 of an adjacent Cu1 anion. The di­ethyl­ammonium cation that includes N3 has both a bifurcated hydrogen bond to Cl3 and Cl4 (Cu1) and a single donor hydrogen bond to Cl7 (Cu3). The hydrogen atoms on N4 are donor atoms of bifurcated hydrogen bonds to Cl5/Cl6 on Cu2 and Cl7/Cl8 on Cu3. The ultimate result of this prolific hydrogen-bond bridging of [CuCl_4_]^2−^ anions is a two-dimensional sheet extending parallel to the *ab* plane (Fig. 2 ▸). Inspection of this plane along the crystallographic *a* axis reveals a gentle corrugation of the sheet (Fig. 2 ▸ *b*). This hydrogen-bonded sheet is likely the driving force for crystallization (Desiraju, 2002 ▸).

## Database survey   

There are 59 structures that incorporate the bis-di­ethyl­ammonium ligand moiety with a tetra­chlorido­cuprate complex (Groom & Allen 2014 ▸; CSD Version 5.36). Of those 59 structures, 23 incorporate bridging chloride ligands, while 36 have independent tetra­chlorido­cuprate complexes present. Thirteen structures incorporate the bis-ethyl­ammonium ligand as a linear structure as presented in this manuscript. In addition, of the 59 structures, eleven show the tetra­chlorido­cuprate complex adopting a distorted square-planar geometry as presented in complex (I).

## Synthesis and crystallization   

**Figure 3:**
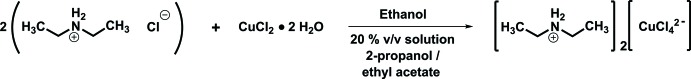
The synthetic scheme.

The synthetic procedure is outlined in Fig. 3 ▸.

**General Procedure**: Bis-di­ethyl­ammonium tetra­chlorido­cuprate was synthesized according to literature procedures (Choi & Larrabee, 1989 ▸). Reagents and solvents used were purchased from commercial sources (Sigma-Aldrich and Fisher Scientific). A Perkin Elmer FT–ATR spectrometer was used to collect IR spectra with three scans from 200 nm to 800 nm at a resolution of 1 cm^−1^. The melting point was recorded on a Fluka Mel-Temp melting point apparatus (Electrothermal) equipped with 51 II thermometer.

**Synthesis of bis-di­ethyl­ammonium tetra­chlorido­cuprate**: Di­ethyl­ammonium hydro­chloride (2.22 g, 20.3 mmol) was dissolved in 15 mL of 2-propanol to afford a clear solution. Copper(II) chloride dihydrate (1.75 g, 10.1 mmol) was dissolved in 3 ml ethanol producing a dark green solution. Both solutions were mixed, generating a brownish-black colored product that was heated in a water bath for 3 min. Upon removal from the water bath, a 10 ml solution of 20% *v*/*v* 2-propanol and ethyl acetate was added to the mixture. The mixture was placed in an ice bath, which gave a bright-green precipitate. The precipitate was filtered, washed with three 10 ml aliquots of ethyl acetate, then air dried to produce the desired product as a bright green thermochromic solid (1.72 g, 48%). M.p. 359.2–359.5 K.

**Thermochromic properties:** Green-colored solid at temperatures lower than 327 K and bright-yellow colored solid at temperatures greater than 328 K.

FT–ATR (solid): *v* (cm^−1^) = 3060 (*s*), 3009 (*s*), 2986 (*br*), 2956 (*s*), 2852 (*s*), 2826 (*s*). Green crystals for complex (I) were obtained by slow diffusion of diethyl ether into a solution of bis-di­ethyl­ammonium tetra­chlorido­cuprate made in methanol.

## Refinement   

Details of the refinement are found in Table 4 ▸. All non-hydrogen atoms were refined with anisotropic atomic displacement parameters. Hydrogen atoms bonded to carbon were included in geometrically calculated positions with *U*_iso_(*H*) = 1.2*U*_eq_(C_methyl­ene_) and 1.5*U*_eq_(C_meth­yl_). Methyl groups were allowed a torsional degree of freedom and C—H distances were set to 0.99 Å (methyl­ene) and 0.98 Å (meth­yl). Ammonium hydrogen atoms were located in difference Fourier maps and refined freely. The structure was refined as an inversion twin, with a 0.52:0.48 twin ratio. Because this ratio is close to 0.5, data were inspected carefully for signs of missed inversion symmetry; no higher symmetry was found. One reflection (0 0 1) was obscured by the beamstop and was omitted from the refinement.

## Supplementary Material

Crystal structure: contains datablock(s) I. DOI: 10.1107/S2056989015023348/pk2565sup1.cif

Structure factors: contains datablock(s) I. DOI: 10.1107/S2056989015023348/pk2565Isup2.hkl

Supporting information file. DOI: 10.1107/S2056989015023348/pk2565Isup3.pdf

CCDC reference: 1440683

Additional supporting information:  crystallographic information; 3D view; checkCIF report

## Figures and Tables

**Table 1:** Selected geometric parameters (Å, °)

Cu1—Cl2	2.2474 (7)	Cu2—Cl6^i^	2.2689 (6)
Cu1—Cl1	2.2598 (7)	Cu2—Cl6	2.2689 (6)
Cu1—Cl3	2.2620 (7)	Cu3—Cl8	2.2475 (7)
Cu1—Cl4	2.2702 (7)	Cu3—Cl8^ii^	2.2475 (7)
Cu2—Cl5	2.2644 (6)	Cu3—Cl7	2.2481 (6)
Cu2—Cl5^i^	2.2644 (6)	Cu3—Cl7^ii^	2.2481 (6)
			
Cl2—Cu1—Cl1	93.20 (3)	Cl5—Cu2—Cl6	90.34 (2)
Cl2—Cu1—Cl3	92.13 (3)	Cl5^i^—Cu2—Cl6	89.66 (2)
Cl1—Cu1—Cl3	161.22 (3)	Cl6^i^—Cu2—Cl6	179.81 (4)
Cl2—Cu1—Cl4	160.16 (3)	Cl8—Cu3—Cl8^ii^	146.10 (4)
Cl1—Cu1—Cl4	90.46 (3)	Cl8—Cu3—Cl7	94.66 (2)
Cl3—Cu1—Cl4	90.60 (3)	Cl8^ii^—Cu3—Cl7	95.17 (2)
Cl5—Cu2—Cl5^i^	176.78 (4)	Cl8—Cu3—Cl7^ii^	95.17 (2)
Cl5—Cu2—Cl6^i^	89.66 (2)	Cl8^ii^—Cu3—Cl7^ii^	94.66 (2)
Cl5^i^—Cu2—Cl6^i^	90.34 (2)	Cl7—Cu3—Cl7^ii^	145.83 (4)

Symmetry codes: (i) 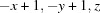; (ii) 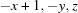.

**Table 2:** Mean plane deviations for [CuCl_4_]^2−^ anions (Å) *Because these pairs of atoms are symmetry related by a twofold axis, deviations are identical.

Atom	Deviation	Atom	Deviation	Atom	Deviation
Cu1	0.0091 (4)	Cu2	0.0239 (5)	Cu3	−0.0021 (5)
Cl1	0.3740 (4)	Cl5/Cl5^i^*	−0.0397 (6)	Cl7/Cl7^ii^*	0.6583 (5)
Cl2	−0.3745 (4)	Cl6/Cl6^i^*	0.0277 (6)	Cl8/Cl8^ii^*	−0.6573 (6)
Cl3	0.3769 (4)				
Cl4	−0.3854 (4)				
					
r.m.s. deviation	0.3379		0.0324		0.5883

Symmetry codes: (i) −*x* + 1, −*y* + 1, *z*; (ii) −*x* + 1, −*y*, *z*.

**Table 3:** Hydrogen-bond geometry (Å, °)

*D*—H⋯*A*	*D*—H	H⋯*A*	*D*⋯*A*	*D*—H⋯*A*
N1—H1*A*⋯Cl5^iii^	0.84 (3)	2.74 (3)	3.316 (2)	128 (2)
N1—H1*A*⋯Cl6^iv^	0.84 (3)	2.53 (3)	3.323 (2)	158 (2)
N1—H1*B*⋯Cl1	0.96 (3)	2.23 (3)	3.192 (2)	178 (3)
N2—H2*C*⋯Cl2^v^	0.84 (3)	2.53 (3)	3.316 (2)	155 (3)
N2—H2*C*⋯Cl3^v^	0.84 (3)	2.72 (3)	3.319 (3)	129 (2)
N2—H2*D*⋯Cl4	0.91 (3)	2.28 (3)	3.180 (2)	171 (3)
N3—H3*C*⋯Cl7	0.82 (3)	2.39 (3)	3.209 (3)	176 (3)
N3—H3*D*⋯Cl3	0.92 (3)	2.53 (3)	3.383 (2)	154 (3)
N3—H3*D*⋯Cl4	0.92 (3)	2.56 (3)	3.198 (3)	127 (2)
N4—H4*D*⋯Cl7^vi^	0.82 (3)	2.93 (3)	3.374 (3)	116 (2)
N4—H4*D*⋯Cl8^vii^	0.82 (3)	2.40 (3)	3.202 (3)	167 (2)
N4—H4*E*⋯Cl5	0.86 (3)	2.47 (3)	3.283 (2)	159 (3)
N4—H4*E*⋯Cl6	0.86 (3)	2.75 (3)	3.311 (3)	125 (2)

Symmetry codes: (iii) 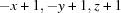; (iv) 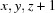; (v) 
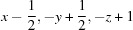
; (vi) 
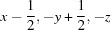
; (vii) 
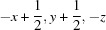
.

**Table 4:** Experimental details

Crystal data	
Chemical formula	(C_4_H_12_N)[Cl_4_Cu]
*M*_r_	353.63
Crystal system, space group	Orthorhombic, *P*2_1_2_1_2
Temperature (K)	120
*a*, *b*, *c* (Å)	14.8766 (13), 29.903 (3), 7.3102 (6)
*V* (Å^3^)	3252.0 (5)
*Z*	8
Radiation type	Mo *K*α
μ (mm^−1^)	1.98
Crystal size (mm)	0.20 × 0.13 × 0.09

Data collection	
Diffractometer	Bruker APEXII
Absorption correction	Multi-scan (*SADABS*; Bruker, 2014 ▸)
*T*_min_, *T*_max_	0.868, 1.000
No. of measured, independent and observed [*I* > 2σ(*I*)] reflections	67459, 6699, 6278
*R*_int_	0.040
(sin θ/λ)_max_ (Å^−1^)	0.627

Refinement	
*R*[*F* ^2^ > 2σ(*F* ^2^)], *wR*(*F* ^2^), *S*	0.020, 0.043, 1.10
No. of reflections	6699
No. of parameters	313
H-atom treatment	H atoms treated by a mixture of independent and constrained refinement
Δρ_max_, Δρ_min_ (e Å^−3^)	0.34, −0.22
Absolute structure	Refined as an inversion twin
Absolute structure parameter	0.523 (10)

Computer programs: *APEX2* and *SAINT* (Bruker, 2014 ▸), *SHELXS* (Sheldrick, 2008 ▸), *SHELXL2014* (Sheldrick, 2015 ▸), *OLEX2* (Dolomanov *et al.*, 2009 ▸) and *publCIF* (Westrip, 2010 ▸).

## supplementary crystallographic information

### Crystal data

**Table:**

(C_4_H_12_N)[Cl_4_Cu]	*D*_x_ = 1.445 Mg m^−^^3^
*M**_r_* = 353.63	Mo *K*α radiation, λ = 0.71073 Å
Orthorhombic, *P*2_1_2_1_2	Cell parameters from 9060 reflections
*a* = 14.8766 (13) Å	θ = 2.5–26.4°
*b* = 29.903 (3) Å	µ = 1.98 mm^−^^1^
*c* = 7.3102 (6) Å	*T* = 120 K
*V* = 3252.0 (5) Å^3^	Block, green
*Z* = 8	0.20 × 0.13 × 0.09 mm
*F*(000) = 1464	

### Data collection

**Table:**

Bruker APEXII diffractometer	6699 independent reflections
Radiation source: fine-focus sealed tube	6278 reflections with *I* > 2σ(*I*)
Detector resolution: 8.33 pixels mm^-1^	*R*_int_ = 0.040
combination of ω and φ–scans	θ_max_ = 26.5°, θ_min_ = 1.4°
Absorption correction: multi-scan (*SADABS*; Bruker, 2014)	*h* = −18→18
*T*_min_ = 0.868, *T*_max_ = 1.000	*k* = −37→37
67459 measured reflections	*l* = −9→9

### Refinement

**Table:**

Refinement on *F*^2^	Secondary atom site location: difference Fourier map
Least-squares matrix: full	Hydrogen site location: mixed
*R*[*F*^2^ > 2σ(*F*^2^)] = 0.020	H atoms treated by a mixture of independent and constrained refinement
*wR*(*F*^2^) = 0.043	*w* = 1/[σ^2^(*F*_o_^2^) + (0.0186*P*)^2^ + 0.6108*P*] where *P* = (*F*_o_^2^ + 2*F*_c_^2^)/3
*S* = 1.10	(Δ/σ)_max_ = 0.001
6699 reflections	Δρ_max_ = 0.34 e Å^−^^3^
313 parameters	Δρ_min_ = −0.22 e Å^−^^3^
0 restraints	Absolute structure: Refined as an inversion twin
Primary atom site location: structure-invariant direct methods	Absolute structure parameter: 0.523 (10)

### Special details

**Table:**

Geometry. All e.s.d.'s (except the e.s.d. in the dihedral angle between two l.s. planes) are estimated using the full covariance matrix. The cell e.s.d.'s are taken into account individually in the estimation of e.s.d.'s in distances, angles and torsion angles; correlations between e.s.d.'s in cell parameters are only used when they are defined by crystal symmetry. An approximate (isotropic) treatment of cell e.s.d.'s is used for estimating e.s.d.'s involving l.s. planes.
Refinement. Refined as a 2-component inversion twin.

### Fractional atomic coordinates and isotropic or equivalent isotropic displacement parameters (Å^2^)

**Table:**

	*x*	*y*	*z*	*U*_iso_*/*U*_eq_	
Cu1	0.50594 (2)	0.24256 (2)	0.71725 (4)	0.01615 (8)	
Cl1	0.44388 (5)	0.30272 (2)	0.85530 (10)	0.02928 (17)	
Cl2	0.63668 (5)	0.27793 (2)	0.66434 (11)	0.02476 (15)	
Cl3	0.56951 (4)	0.17463 (2)	0.67495 (10)	0.02242 (14)	
Cl4	0.36734 (4)	0.21319 (2)	0.66777 (11)	0.02605 (16)	
Cu2	0.5000	0.5000	0.17777 (5)	0.01407 (9)	
Cl5	0.36104 (4)	0.53083 (2)	0.16907 (11)	0.02239 (14)	
Cl6	0.43871 (4)	0.43052 (2)	0.17828 (10)	0.02188 (14)	
Cu3	0.5000	0.0000	0.23370 (6)	0.01802 (10)	
Cl7	0.57118 (4)	0.06253 (2)	0.32404 (10)	0.02721 (16)	
Cl8	0.37459 (5)	0.03573 (2)	0.14408 (10)	0.02458 (15)	
N1	0.59486 (16)	0.37538 (8)	0.9442 (3)	0.0175 (5)	
H1A	0.5680 (18)	0.3946 (8)	1.007 (4)	0.010 (7)*	
H1B	0.549 (2)	0.3539 (11)	0.914 (4)	0.040 (10)*	
C1	0.6240 (2)	0.33898 (9)	1.2396 (4)	0.0286 (7)	
H1C	0.5724	0.3196	1.2146	0.043*	
H1D	0.6042	0.3651	1.3098	0.043*	
H1E	0.6690	0.3224	1.3102	0.043*	
C2	0.66469 (18)	0.35417 (9)	1.0618 (4)	0.0211 (6)	
H2A	0.7134	0.3759	1.0865	0.025*	
H2B	0.6911	0.3282	0.9972	0.025*	
C3	0.62953 (18)	0.39622 (8)	0.7727 (4)	0.0194 (6)	
H3A	0.6612	0.3734	0.6989	0.023*	
H3B	0.6731	0.4201	0.8038	0.023*	
C4	0.55385 (19)	0.41575 (9)	0.6626 (4)	0.0274 (6)	
H4A	0.5779	0.4292	0.5506	0.041*	
H4B	0.5231	0.4387	0.7349	0.041*	
H4C	0.5111	0.3921	0.6306	0.041*	
N2	0.24950 (17)	0.29865 (7)	0.5700 (3)	0.0179 (5)	
H2C	0.209 (2)	0.2864 (10)	0.506 (4)	0.026 (9)*	
H2D	0.288 (2)	0.2763 (11)	0.604 (5)	0.040 (10)*	
C5	0.3393 (2)	0.31134 (10)	0.2914 (4)	0.0360 (8)	
H5A	0.2911	0.2986	0.2164	0.054*	
H5B	0.3811	0.2876	0.3275	0.054*	
H5C	0.3716	0.3341	0.2206	0.054*	
C6	0.29959 (19)	0.33250 (9)	0.4599 (4)	0.0232 (6)	
H6A	0.3482	0.3456	0.5352	0.028*	
H6B	0.2583	0.3569	0.4236	0.028*	
C7	0.20750 (19)	0.31699 (8)	0.7396 (4)	0.0215 (6)	
H7A	0.1604	0.3389	0.7060	0.026*	
H7B	0.2537	0.3328	0.8127	0.026*	
C8	0.1665 (2)	0.28018 (9)	0.8528 (4)	0.0287 (7)	
H8A	0.1200	0.2649	0.7814	0.043*	
H8B	0.1396	0.2930	0.9634	0.043*	
H8C	0.2133	0.2587	0.8874	0.043*	
N3	0.40456 (16)	0.12593 (8)	0.4264 (3)	0.0175 (5)	
H3C	0.446 (2)	0.1091 (9)	0.396 (4)	0.022 (8)*	
H3D	0.434 (2)	0.1453 (10)	0.504 (5)	0.035 (9)*	
C9	0.4391 (2)	0.17652 (10)	0.1694 (5)	0.0391 (8)	
H9A	0.4126	0.1933	0.0677	0.059*	
H9B	0.4839	0.1555	0.1218	0.059*	
H9C	0.4681	0.1973	0.2545	0.059*	
C10	0.36649 (19)	0.15100 (9)	0.2681 (4)	0.0240 (6)	
H10A	0.3200	0.1721	0.3120	0.029*	
H10B	0.3375	0.1298	0.1824	0.029*	
C11	0.33663 (19)	0.10125 (9)	0.5365 (4)	0.0232 (6)	
H11A	0.3053	0.0794	0.4574	0.028*	
H11B	0.2914	0.1225	0.5840	0.028*	
C12	0.3800 (2)	0.07722 (11)	0.6935 (4)	0.0391 (8)	
H12A	0.4234	0.0554	0.6464	0.059*	
H12B	0.3339	0.0617	0.7650	0.059*	
H12C	0.4112	0.0989	0.7717	0.059*	
N4	0.25278 (17)	0.45225 (7)	−0.0543 (3)	0.0170 (5)	
H4D	0.2139 (19)	0.4704 (9)	−0.082 (4)	0.014 (8)*	
H4E	0.289 (2)	0.4666 (10)	0.016 (4)	0.029 (9)*	
C13	0.1651 (2)	0.43942 (10)	0.2256 (4)	0.0336 (8)	
H13A	0.1388	0.4163	0.3039	0.050*	
H13B	0.1178	0.4598	0.1841	0.050*	
H13C	0.2103	0.4562	0.2950	0.050*	
C14	0.20883 (19)	0.41794 (9)	0.0629 (4)	0.0215 (6)	
H14A	0.2543	0.3960	0.1048	0.026*	
H14B	0.1630	0.4017	−0.0093	0.026*	
C15	0.29877 (19)	0.43473 (9)	−0.2199 (4)	0.0206 (6)	
H15A	0.2558	0.4169	−0.2931	0.025*	
H15B	0.3487	0.4148	−0.1827	0.025*	
C16	0.3352 (2)	0.47240 (9)	−0.3351 (4)	0.0288 (7)	
H16A	0.3787	0.4897	−0.2635	0.043*	
H16B	0.2857	0.4919	−0.3731	0.043*	
H16C	0.3648	0.4601	−0.4437	0.043*	

### Atomic displacement parameters (Å^2^)

**Table:**

	*U*^11^	*U*^22^	*U*^33^	*U*^12^	*U*^13^	*U*^23^
Cu1	0.01612 (17)	0.01610 (13)	0.01623 (14)	0.00025 (13)	−0.00063 (14)	−0.00147 (11)
Cl1	0.0210 (3)	0.0272 (3)	0.0396 (4)	−0.0021 (3)	0.0045 (3)	−0.0171 (3)
Cl2	0.0235 (4)	0.0192 (3)	0.0315 (4)	−0.0043 (3)	0.0095 (3)	−0.0028 (3)
Cl3	0.0173 (3)	0.0171 (3)	0.0329 (4)	0.0018 (2)	−0.0008 (3)	−0.0020 (3)
Cl4	0.0167 (3)	0.0195 (3)	0.0420 (4)	0.0020 (3)	−0.0057 (3)	−0.0080 (3)
Cu2	0.0138 (2)	0.01348 (18)	0.01494 (19)	0.00051 (18)	0.000	0.000
Cl5	0.0150 (3)	0.0156 (3)	0.0366 (4)	0.0012 (2)	−0.0013 (3)	−0.0009 (3)
Cl6	0.0176 (3)	0.0145 (3)	0.0336 (4)	−0.0004 (2)	−0.0002 (3)	0.0003 (3)
Cu3	0.0163 (2)	0.0211 (2)	0.0167 (2)	−0.0010 (2)	0.000	0.000
Cl7	0.0163 (3)	0.0299 (3)	0.0353 (4)	−0.0014 (3)	0.0005 (3)	−0.0148 (3)
Cl8	0.0244 (4)	0.0192 (3)	0.0301 (4)	−0.0011 (3)	−0.0110 (3)	0.0005 (3)
N1	0.0179 (12)	0.0165 (11)	0.0180 (12)	0.0017 (10)	0.0007 (10)	−0.0014 (10)
C1	0.0357 (18)	0.0244 (14)	0.0257 (16)	0.0045 (13)	−0.0027 (14)	0.0024 (12)
C2	0.0201 (15)	0.0195 (13)	0.0237 (15)	0.0045 (11)	−0.0048 (12)	−0.0033 (11)
C3	0.0209 (14)	0.0177 (12)	0.0197 (14)	0.0010 (11)	0.0029 (12)	−0.0016 (11)
C4	0.0304 (16)	0.0282 (14)	0.0237 (14)	0.0006 (13)	−0.0008 (14)	0.0042 (12)
N2	0.0162 (12)	0.0170 (11)	0.0206 (12)	−0.0007 (10)	0.0002 (10)	−0.0025 (10)
C5	0.042 (2)	0.0366 (16)	0.0295 (18)	−0.0040 (15)	0.0156 (15)	0.0008 (14)
C6	0.0229 (15)	0.0222 (14)	0.0244 (15)	−0.0025 (12)	0.0024 (12)	0.0023 (12)
C7	0.0227 (15)	0.0207 (13)	0.0209 (14)	0.0027 (11)	0.0015 (12)	−0.0053 (11)
C8	0.0299 (16)	0.0290 (14)	0.0271 (16)	0.0044 (13)	0.0086 (14)	0.0009 (13)
N3	0.0147 (12)	0.0180 (12)	0.0198 (12)	0.0004 (10)	0.0008 (10)	−0.0011 (10)
C9	0.0379 (19)	0.0448 (17)	0.0347 (18)	−0.0064 (15)	−0.0010 (17)	0.0159 (16)
C10	0.0227 (15)	0.0254 (13)	0.0237 (15)	0.0007 (12)	−0.0074 (13)	0.0031 (12)
C11	0.0201 (15)	0.0245 (14)	0.0251 (15)	−0.0035 (12)	0.0050 (12)	−0.0008 (12)
C12	0.0382 (19)	0.0482 (19)	0.0310 (18)	−0.0069 (15)	0.0045 (15)	0.0184 (16)
N4	0.0147 (13)	0.0158 (11)	0.0204 (12)	−0.0008 (10)	−0.0018 (10)	−0.0014 (10)
C13	0.043 (2)	0.0294 (15)	0.0285 (17)	−0.0068 (14)	0.0100 (15)	0.0024 (13)
C14	0.0207 (15)	0.0183 (13)	0.0255 (15)	−0.0030 (11)	0.0001 (12)	0.0043 (11)
C15	0.0183 (14)	0.0217 (12)	0.0217 (14)	0.0024 (11)	0.0020 (12)	−0.0046 (11)
C16	0.0289 (16)	0.0312 (15)	0.0264 (15)	0.0032 (13)	0.0082 (14)	0.0031 (14)

### Geometric parameters (Å, º)

**Table:**

Cu1—Cl2	2.2474 (7)	C7—C8	1.506 (4)
Cu1—Cl1	2.2598 (7)	C7—H7A	0.9900
Cu1—Cl3	2.2620 (7)	C7—H7B	0.9900
Cu1—Cl4	2.2702 (7)	C8—H8A	0.9800
Cu2—Cl5	2.2644 (6)	C8—H8B	0.9800
Cu2—Cl5^i^	2.2644 (6)	C8—H8C	0.9800
Cu2—Cl6^i^	2.2689 (6)	N3—C11	1.488 (3)
Cu2—Cl6	2.2689 (6)	N3—C10	1.491 (3)
Cu3—Cl8	2.2475 (7)	N3—H3C	0.82 (3)
Cu3—Cl8^ii^	2.2475 (7)	N3—H3D	0.92 (3)
Cu3—Cl7	2.2481 (6)	C9—C10	1.507 (4)
Cu3—Cl7^ii^	2.2481 (6)	C9—H9A	0.9800
N1—C2	1.490 (3)	C9—H9B	0.9800
N1—C3	1.492 (3)	C9—H9C	0.9800
N1—H1A	0.84 (3)	C10—H10A	0.9900
N1—H1B	0.96 (3)	C10—H10B	0.9900
C1—C2	1.504 (4)	C11—C12	1.500 (4)
C1—H1C	0.9800	C11—H11A	0.9900
C1—H1D	0.9800	C11—H11B	0.9900
C1—H1E	0.9800	C12—H12A	0.9800
C2—H2A	0.9900	C12—H12B	0.9800
C2—H2B	0.9900	C12—H12C	0.9800
C3—C4	1.502 (4)	N4—C15	1.486 (3)
C3—H3A	0.9900	N4—C14	1.488 (3)
C3—H3B	0.9900	N4—H4D	0.82 (3)
C4—H4A	0.9800	N4—H4E	0.86 (3)
C4—H4B	0.9800	C13—C14	1.500 (4)
C4—H4C	0.9800	C13—H13A	0.9800
N2—C6	1.492 (3)	C13—H13B	0.9800
N2—C7	1.493 (3)	C13—H13C	0.9800
N2—H2C	0.84 (3)	C14—H14A	0.9900
N2—H2D	0.91 (3)	C14—H14B	0.9900
C5—C6	1.506 (4)	C15—C16	1.507 (4)
C5—H5A	0.9800	C15—H15A	0.9900
C5—H5B	0.9800	C15—H15B	0.9900
C5—H5C	0.9800	C16—H16A	0.9800
C6—H6A	0.9900	C16—H16B	0.9800
C6—H6B	0.9900	C16—H16C	0.9800
			
Cl2—Cu1—Cl1	93.20 (3)	N2—C7—H7B	109.5
Cl2—Cu1—Cl3	92.13 (3)	C8—C7—H7B	109.5
Cl1—Cu1—Cl3	161.22 (3)	H7A—C7—H7B	108.0
Cl2—Cu1—Cl4	160.16 (3)	C7—C8—H8A	109.5
Cl1—Cu1—Cl4	90.46 (3)	C7—C8—H8B	109.5
Cl3—Cu1—Cl4	90.60 (3)	H8A—C8—H8B	109.5
Cl5—Cu2—Cl5^i^	176.78 (4)	C7—C8—H8C	109.5
Cl5—Cu2—Cl6^i^	89.66 (2)	H8A—C8—H8C	109.5
Cl5^i^—Cu2—Cl6^i^	90.34 (2)	H8B—C8—H8C	109.5
Cl5—Cu2—Cl6	90.34 (2)	C11—N3—C10	114.3 (2)
Cl5^i^—Cu2—Cl6	89.66 (2)	C11—N3—H3C	110 (2)
Cl6^i^—Cu2—Cl6	179.81 (4)	C10—N3—H3C	112 (2)
Cl8—Cu3—Cl8^ii^	146.10 (4)	C11—N3—H3D	108 (2)
Cl8—Cu3—Cl7	94.66 (2)	C10—N3—H3D	110 (2)
Cl8^ii^—Cu3—Cl7	95.17 (2)	H3C—N3—H3D	102 (3)
Cl8—Cu3—Cl7^ii^	95.17 (2)	C10—C9—H9A	109.5
Cl8^ii^—Cu3—Cl7^ii^	94.66 (2)	C10—C9—H9B	109.5
Cl7—Cu3—Cl7^ii^	145.83 (4)	H9A—C9—H9B	109.5
C2—N1—C3	114.9 (2)	C10—C9—H9C	109.5
C2—N1—H1A	108.0 (18)	H9A—C9—H9C	109.5
C3—N1—H1A	109.8 (18)	H9B—C9—H9C	109.5
C2—N1—H1B	109.9 (19)	N3—C10—C9	110.7 (2)
C3—N1—H1B	109 (2)	N3—C10—H10A	109.5
H1A—N1—H1B	104 (3)	C9—C10—H10A	109.5
C2—C1—H1C	109.5	N3—C10—H10B	109.5
C2—C1—H1D	109.5	C9—C10—H10B	109.5
H1C—C1—H1D	109.5	H10A—C10—H10B	108.1
C2—C1—H1E	109.5	N3—C11—C12	111.0 (2)
H1C—C1—H1E	109.5	N3—C11—H11A	109.4
H1D—C1—H1E	109.5	C12—C11—H11A	109.4
N1—C2—C1	110.3 (2)	N3—C11—H11B	109.4
N1—C2—H2A	109.6	C12—C11—H11B	109.4
C1—C2—H2A	109.6	H11A—C11—H11B	108.0
N1—C2—H2B	109.6	C11—C12—H12A	109.5
C1—C2—H2B	109.6	C11—C12—H12B	109.5
H2A—C2—H2B	108.1	H12A—C12—H12B	109.5
N1—C3—C4	110.7 (2)	C11—C12—H12C	109.5
N1—C3—H3A	109.5	H12A—C12—H12C	109.5
C4—C3—H3A	109.5	H12B—C12—H12C	109.5
N1—C3—H3B	109.5	C15—N4—C14	115.4 (2)
C4—C3—H3B	109.5	C15—N4—H4D	111 (2)
H3A—C3—H3B	108.1	C14—N4—H4D	106.8 (19)
C3—C4—H4A	109.5	C15—N4—H4E	112 (2)
C3—C4—H4B	109.5	C14—N4—H4E	106 (2)
H4A—C4—H4B	109.5	H4D—N4—H4E	105 (3)
C3—C4—H4C	109.5	C14—C13—H13A	109.5
H4A—C4—H4C	109.5	C14—C13—H13B	109.5
H4B—C4—H4C	109.5	H13A—C13—H13B	109.5
C6—N2—C7	114.1 (2)	C14—C13—H13C	109.5
C6—N2—H2C	110 (2)	H13A—C13—H13C	109.5
C7—N2—H2C	109 (2)	H13B—C13—H13C	109.5
C6—N2—H2D	109 (2)	N4—C14—C13	110.6 (2)
C7—N2—H2D	108 (2)	N4—C14—H14A	109.5
H2C—N2—H2D	106 (3)	C13—C14—H14A	109.5
C6—C5—H5A	109.5	N4—C14—H14B	109.5
C6—C5—H5B	109.5	C13—C14—H14B	109.5
H5A—C5—H5B	109.5	H14A—C14—H14B	108.1
C6—C5—H5C	109.5	N4—C15—C16	110.9 (2)
H5A—C5—H5C	109.5	N4—C15—H15A	109.5
H5B—C5—H5C	109.5	C16—C15—H15A	109.5
N2—C6—C5	110.6 (2)	N4—C15—H15B	109.5
N2—C6—H6A	109.5	C16—C15—H15B	109.5
C5—C6—H6A	109.5	H15A—C15—H15B	108.0
N2—C6—H6B	109.5	C15—C16—H16A	109.5
C5—C6—H6B	109.5	C15—C16—H16B	109.5
H6A—C6—H6B	108.1	H16A—C16—H16B	109.5
N2—C7—C8	110.9 (2)	C15—C16—H16C	109.5
N2—C7—H7A	109.5	H16A—C16—H16C	109.5
C8—C7—H7A	109.5	H16B—C16—H16C	109.5
			
C3—N1—C2—C1	173.6 (2)	C11—N3—C10—C9	177.6 (2)
C2—N1—C3—C4	178.9 (2)	C10—N3—C11—C12	−179.6 (2)
C7—N2—C6—C5	−179.7 (2)	C15—N4—C14—C13	179.8 (2)
C6—N2—C7—C8	−174.1 (2)	C14—N4—C15—C16	176.0 (2)

Symmetry codes: (i) −*x*+1, −*y*+1, *z*; (ii) −*x*+1, −*y*, *z*.

### Hydrogen-bond geometry (Å, º)

**Table:**

*D*—H···*A*	*D*—H	H···*A*	*D*···*A*	*D*—H···*A*
N1—H1*A*···Cl5^iii^	0.84 (3)	2.74 (3)	3.316 (2)	128 (2)
N1—H1*A*···Cl6^iv^	0.84 (3)	2.53 (3)	3.323 (2)	158 (2)
N1—H1*B*···Cl1	0.96 (3)	2.23 (3)	3.192 (2)	178 (3)
N2—H2*C*···Cl2^v^	0.84 (3)	2.53 (3)	3.316 (2)	155 (3)
N2—H2*C*···Cl3^v^	0.84 (3)	2.72 (3)	3.319 (3)	129 (2)
N2—H2*D*···Cl4	0.91 (3)	2.28 (3)	3.180 (2)	171 (3)
N3—H3*C*···Cl7	0.82 (3)	2.39 (3)	3.209 (3)	176 (3)
N3—H3*D*···Cl3	0.92 (3)	2.53 (3)	3.383 (2)	154 (3)
N3—H3*D*···Cl4	0.92 (3)	2.56 (3)	3.198 (3)	127 (2)
N4—H4*D*···Cl7^vi^	0.82 (3)	2.93 (3)	3.374 (3)	116 (2)
N4—H4*D*···Cl8^vii^	0.82 (3)	2.40 (3)	3.202 (3)	167 (2)
N4—H4*E*···Cl5	0.86 (3)	2.47 (3)	3.283 (2)	159 (3)
N4—H4*E*···Cl6	0.86 (3)	2.75 (3)	3.311 (3)	125 (2)

Symmetry codes: (iii) −*x*+1, −*y*+1, *z*+1; (iv) *x*, *y*, *z*+1; (v) *x*−1/2, −*y*+1/2, −*z*+1; (vi) *x*−1/2, −*y*+1/2, −*z*; (vii) −*x*+1/2, *y*+1/2, −*z*.
