# Supplementary material for: Crystal structure of the thermochromic bis­(di­ethyl­ammonium) tetra­chlorido­cuprate(II) complex
Source: Acta Crystallogr E Crystallogr Commun. 2016 Jan 1;72(Pt 1):40–3. doi: 10.1107/S2056989015023348 (PMC4704760; doi:10.1107/S2056989015023348)
Supplement: Supplementary file 3 [file e-72-00040-Isup3.pdf]

1) Please add the proper anion charges to the scheme to balance the cation charges.

**Author Response: Our apologies for this oversight. The scheme is now corrected to reflect a 4+ and 4– charge balance as advised.**

2) Abstract: How does a 'pinwheel-like geometry' extend along the stated direction? Is it a stack of pinwheels, or a chain of some sort? It looks to me as though the pinwheels about each other in the ab plane rather than just in one 'direction'.

**Author response: The abstract has been thoroughly revised and the information about pinwheel connectivity now modified.**

3) Abstract: Since there are three independent [CuCl4]2- units (or rather one whole and two halves), do they all generate pinwheels ? If so then this needs to be stated as such.

**Author response: Please see #2 above.**

4) Abstract: What is meant by 'parallel to the z axis'? Does it mean 'c-axis', and if it does, what exactly is parallel to c ? I'm guessing that it means the 2-fold rotation axis is parallel to c, but there is no need to state this explicitly as that is merely a consequence of the space group, which by definition has a 2-fold along c.

**Author response: Thanks for this input, as above, the abstract has been revised.**

5) General: I think the Abstract ought to be re-written so that it forms a coherent whole. As it stands, it is too disjointed. You could get an idea of how to write the Abstract by reading other Acta E structure descriptions.

**Author response: We thank you for the suggestion; we have taken this into consideration and revised the abstract completely.**

6) Chemical context: What is meant by 'compounds lattice'?

**Author response: Thank you for pointing this out, the wording has been changed to now state "lattice structure". Hopefully this provides better clarity.**

7) Structural commentary: "A complete list of these interactions is refined and ..." Is the list 'refined' in Table 1? Please rewrite.

**Author response: the discussion has been rephrased as requested.**

8) Structural commentary: It ought to be possible to calculate standard uncertainties for the RMS deviations since each individual deviation has an SU value.

**Author response: We have recalculated all values reported in the body text and included s.u.'s as appropriate throughout. Thank you for catching this.**

10) Supramolecular features: I still don't see what is meant by "... pinwheel-like geometry creates a packing motif that extends in the [0 0 1] direction".

**Author response: The Supramolecular Features section has been thoroughly revised. We hope that the modified discussion forms a more reasonable whole.**

11) General: The way you have chosen your asymmetric unit does not seem to be the most convenient for describing the hydrogen bonding. In your asymmetric unit, atom N4 is not positioned so as to hydrogen bond to Cl8. This means that the atom labeled N4 in Fig. 2 is actually a symmetry equivalent of the N4 that is in the actual model. This in turn means that the Figure caption cannot be right. This stuff really needs to be fixed to make it acceptable.

**Author response: The molecular coordinates have been shifted within the unit cell to 1) move them closer to the origin, 2) locate molecular species into more reasonable positions to describe the intermolecular contacts more simply. We hope that the new arrangement is more acceptable. We do realize that this means one of the diethylammonium cations has a center of mass just outside the standard unit cell, but we felt this was preferable rather than having other species further located out-side the standard cell also.**

13) None of the programs used have been referenced. This must be fixed.

**Author response: Thank you and our sincere apologies for this oversight, we have attempted to include all software references as appropriate.**
